# Urinary Exosomal and cell-free DNA Detects Somatic Mutation and Copy Number Alteration in Urothelial Carcinoma of Bladder

**DOI:** 10.1038/s41598-018-32900-6

**Published:** 2018-10-02

**Authors:** Dong Hyeon Lee, Hana Yoon, Sanghui Park, Jeong Seon Kim, Young-Ho Ahn, Kihwan Kwon, Donghwan Lee, Kwang Hyun Kim

**Affiliations:** 1 Department of Urology, Ewha Womans University College of Medicine, Seoul, Korea; 2 Department of Pathology, Ewha Womans University College of Medicine, Seoul, Korea; 3 Department of Molecular Medicine, Ewha Womans University College of Medicine, Seoul, Korea; 4 Department of Cardiology, Ewha Womans University College of Medicine, Seoul, Korea; 5 Department of Statistics, Ewha Womans University, Seoul, Korea

## Abstract

Urothelial bladder carcinoma (UBC) is characterized by a large number of genetic alterations. DNA from urine is a promising source for liquid biopsy in urological malignancies. We aimed to assess the availability of cell-free DNA (cfDNA) and exosomal DNA (exoDNA) in urine as a source for liquid biopsy in UBC. We included 9 patients who underwent surgery for UBC and performed genomic profiling of tumor samples and matched urinary cfDNA and exoDNA. For mutation analysis, deep sequencing was performed for 9 gene targets and shallow whole genome sequencing (sWGS) was used for the detection of copy number variation (CNV). We analyzed whether genetic alteration in tumor samples was reflected in urinary cfDNA or exoDNA. To measure the similarity between copy number profiles of tumor tissue and urinary DNA, the Pearson’s correlation coefficient was calculated. We found 17 somatic mutations in 6 patients. Of the 17 somatic mutations, 14 and 12 were identified by analysis of cfDNA and exoDNA with AFs of 56.2% and 65.6%, respectively. In CNV analysis using sWGS, although the mean depth was 0.6X, we found amplification of MDM2, ERBB2, CCND1 and CCNE1, and deletion of CDKN2A, PTEN and RB1, all known to be frequently altered in UBC. CNV plots of cfDNA and exoDNA showed a similar pattern with those from the tumor samples. Pearson’s correlation coefficients of tumor vs. cfDNA (0.481) and tumor vs. exoDNA (0.412) were higher than that of tumor vs. normal (0.086). We successfully identified somatic mutations and CNV in UBC using urinary cfDNA and exoDNA. Urinary exoDNA could be another source for liquid biopsy. Also, CNV analysis using sWGS is an alternative strategy for liquid biopsy, providing data from the whole genome at a low cost.

## Introduction

Liquid biopsy is a minimally invasive method for identifying genetic alterations in tumors using plasma or other body fluids. There has been an increasing interest in the utility of liquid biopsy alternative to conventional solid biopsy. Liquid biopsy, owing to its less invasive sampling procedure, facilitates genetic profiling of tumors without limiting the frequency of sampling and tumor heterogeneity^1,2^. With technological advances in DNA sequencing, analysis of circulating tumor DNA provides homogenous representation of subclones and microenvironments of tumors, and could serve as a marker for drug susceptibility, prognosis or disease progression in patients with malignancies^3–6^. However, detection of tumor DNA from body fluid is challenging due to the short half-life and low purity of the DNA^7–9^. The analysis of circulating tumor DNA requires a highly sensitive method, and the clinical utility of circulating tumor DNA is usually focused on monitoring disease rather than diagnosing early state disease, which has a low abundance of circulating tumor DNA with few genetic alterations.

Urine is an ideal body fluid for liquid biopsy as it could be collected in a truly non-invasive manner with a relatively reduced limit in volume. Previous studies have reported that cell-free DNA (cfDNA) in circulation passes through glomerular filtration which is known as “trans-renal DNA”^10^. It could be used as a source for circulating tumor DNA and urinary biomarkers^11,12^. Various studies have shown that genomic alteration of non-urological malignancies such as lung cancer, colorectal cancer or pancreatic cancer, can also be identified in urinary cfDNA^10–14^. However, studies on urinary cfDNA are more extensively conducted in urologic malignancies and the origin of urinary cfDNA in urologic malignancies could be both urinary tract cells and trans-renal cfDNA.

Urinary exosomes are also a source of tumor DNA. Exosomes are released from cells and shed into various body fluid including blood and urine. Exosomes are a subset of extracellular vesicles that are potential biomarkers in malignancies because they contain various proteins, lipids and nucleic acids^15^. While most studies on the utilization of nucleic acids in exosomes as biomarkers have focused on miRNAs or mRNAs, exosome contains double-stranded DNA fragments, and genomic alterations in cancer have been identified in exosomal DNA (exoDNA)^16–18^. Circulating exosomes can be isolated from blood and various body fluids such as saliva, breast milk, bile and urine^19^.

In this study, we investigated the availability of urinary cfDNA and exoDNA in liquid biopsy for urinary bladder cancer (UBC). UBC is the second most common urologic malignancy with a large number of genetic alterations^20^. The genomic profiling was performed in 9 patients with UBC and matched urinary cfDNA and exoDNA. To detect somatic mutations, we used targeted deep sequencing of 9 genes that are frequently mutated in UBC. We also analyzed the copy number variation (CNV) in the whole genome region. For clinical applicability, we performed shallow whole genome sequencing (sWGS) and the genome coverage by sWGS was less than 1X in all cases^21^. To detect copy number aberrations, we used the QDNAseq algorithm, which provides high-quality DNA copy number information from data produced by sWGS. This algorithms showed better performance than previous approaches for sWGS analysis, especially in low-quality samples such as DNA from formalin-fixed specimens^21^. CfDNA is fragmented into small sizes and also characterized by low quality and quantity.

## Results

### Patient characteristics and DNA from urine

This study included 9 patients who underwent radical cystectomy for UBC. The clinicopathological characteristics of the patients are summarized in Supplement Table S2. Urinary DNA was extracted in two ways. CfDNA was extracted from the supernatant of urine and exoDNA was extracted from the urinary exosomes. CfDNA was extracted from 2–4 ml and exoDNA was extracted from 10 ml of urine except in one patient with 20 ml of urine. The concentrations of cfDNA and exoDNA were 17.6 ng/ml and 7.9 ng/ml, respectively. In the sWGS process, 92.1% and 92.6% of sequences from cfDNA and exoDNA were mapped in the human genome, suggesting most urinary cfDNA and exoDNA is from the host genome. (Supplement Table S3). While cfDNA was highly fragmented and mostly 150–180 bp in length, exoDNA contained large fragmented DNA compared to the cfDNA (Supplementary Fig. S2).

### Somatic mutation analysis with deep sequencing

For somatic mutation analysis, we performed target capture sequencing of 9 genes that are frequently mutated in UBC. Somatic mutations were identified by tumor and normal matched analysis. We identified 17 somatic mutations in 6 patients (66.7%, 6/9). Seventeen somatic mutation included 6 nonsynonymous SNVs, 3 stop-gain SNVs, 2 frameshift deletions and 6 synonymous SNVs, with a mean allele frequency of 53.6% (12.7–99.7). When we excluded 6 synonymous SNVs, TP53 was the most frequently mutated gene with 3 nonsynonymous and 1 stop-gain SNV.

**Figure 1:**
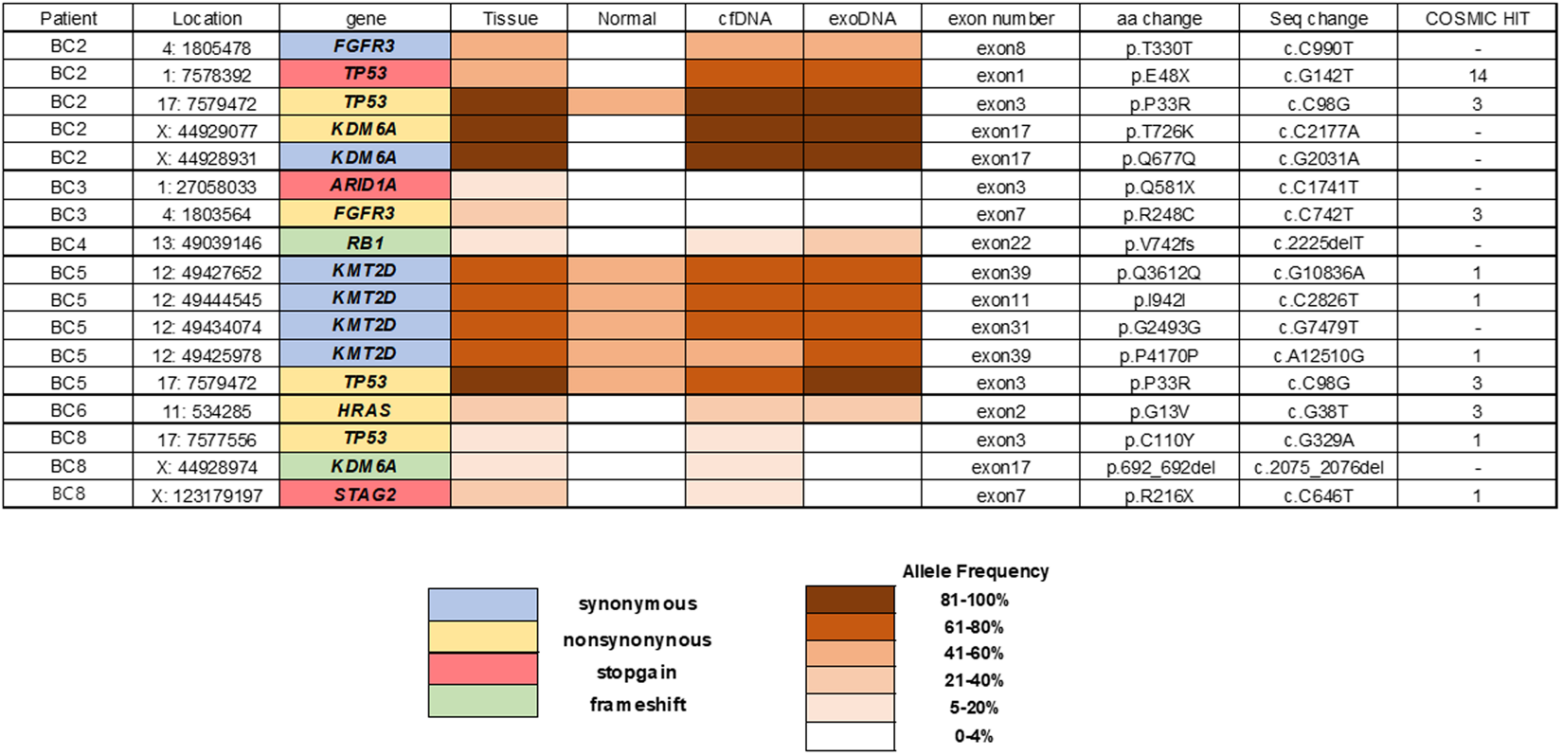
Somatic mutations identified in bladder cancer and genomic profiling in matched urinary cell free DNA and exosomal DNA.

Then, the mutational results of the 9 genes obtained from tumor samples were compared with those from urinary cfDNA and exoDNA. We analyzed the targeted deep sequencing data from urinary cfDNA and exoDNA. While mean depths were 632X and 404X for tumor and paired blood samples, respectively, the mean depths for cfDNA and exoDNA were 1721X and 1648X, respectively (Supplementary Table S1). Of 17 somatic mutations, 14 and 12 were identified by analysis of cfDNA and exoDNA with AFs of 54.5% and 65.6%, respectively (Fig. 1). When we did not exclude SNVs with AF less than 5% in the analysis of cfDNA and exoDNA, one stop-gain SNV in ARID1A (C1714T) with a somatic mutation frequency of 16.8% (58/345) was identified in cfDNA with the allele frequency of 1.8% (21/1177) and exoDNA with the AF of 3.4% (30/897). Also, nonsynonymous SNV in FGFR3 (C742T) with somatic mutation AF of 20.8% (49/235) was identified in exoDNA with AF 1.2% (10/814) (Supplementary Table S4).

**Figure 2:**
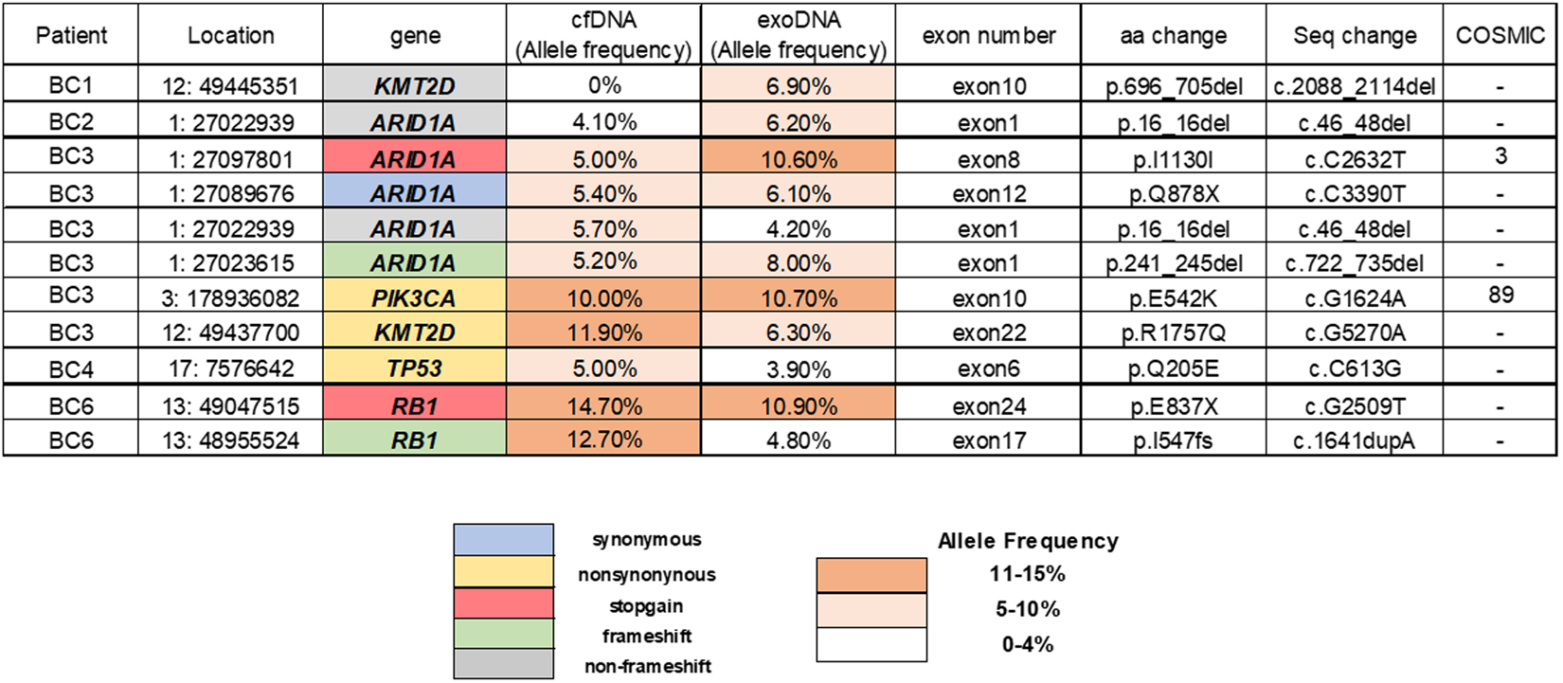
Mutations only identified in urinary cell free DNA and exosomal DNA.

We also identified 11 somatic mutations that were only identified in urinary DNA. CfDNA and exoDNA had 9 and 8 somatic mutation and 6 were identified both in cfDNA and exoDNA (Fig. 2). The mean AFs were 8.6% and 7.2% in cfDNA and exoDNA, respectively. Of the 11 somatic mutations, 7 were clinically significant somatic mutations that included 3 nonsynonymous SNVs, 2 frameshift indels and 2 stop-gain SNVs. Two were also seen in the COSMIC database with frequencies of 3 and 89 (Supplementary Table S5).

### Copy number analysis with sWGS

**Figure 3:**
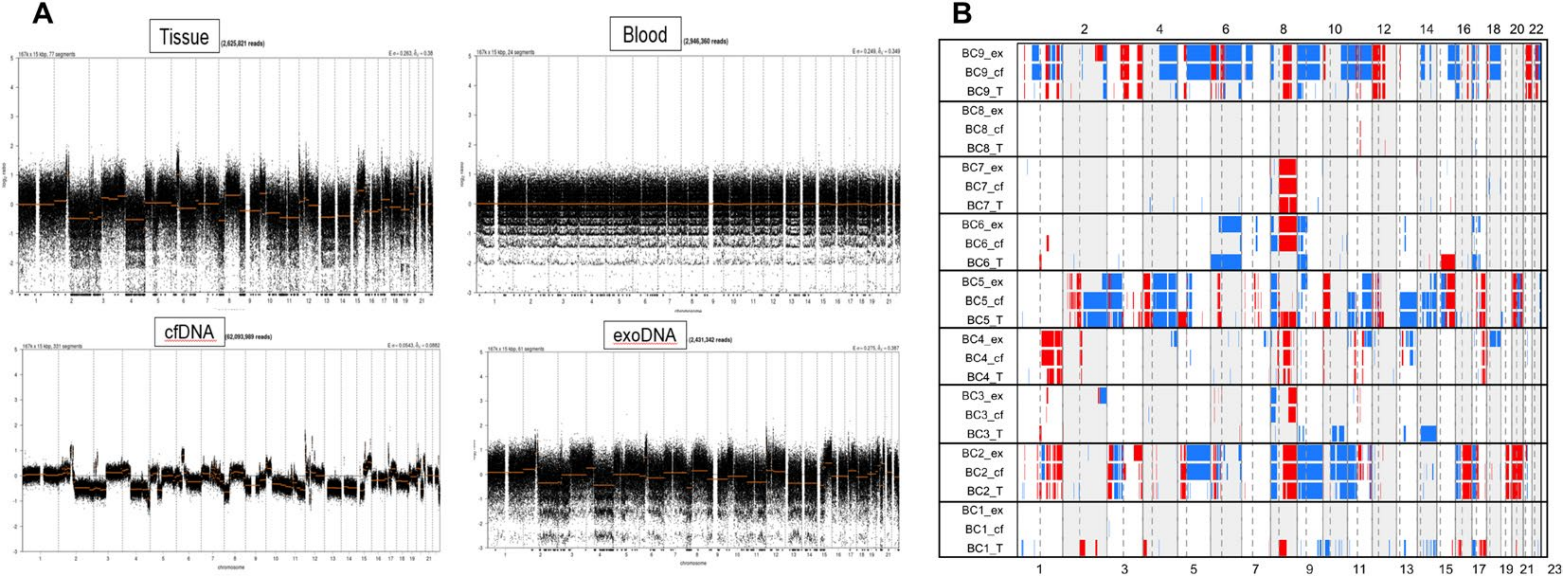
CNV profiles of patients 5 (A) and copy number aberration plot of 9 patients with bladder cancer. Cell free DNA and exosomal DNA samples present similar pattern of copy number aberration with tumor samples.

**Figure 4:**
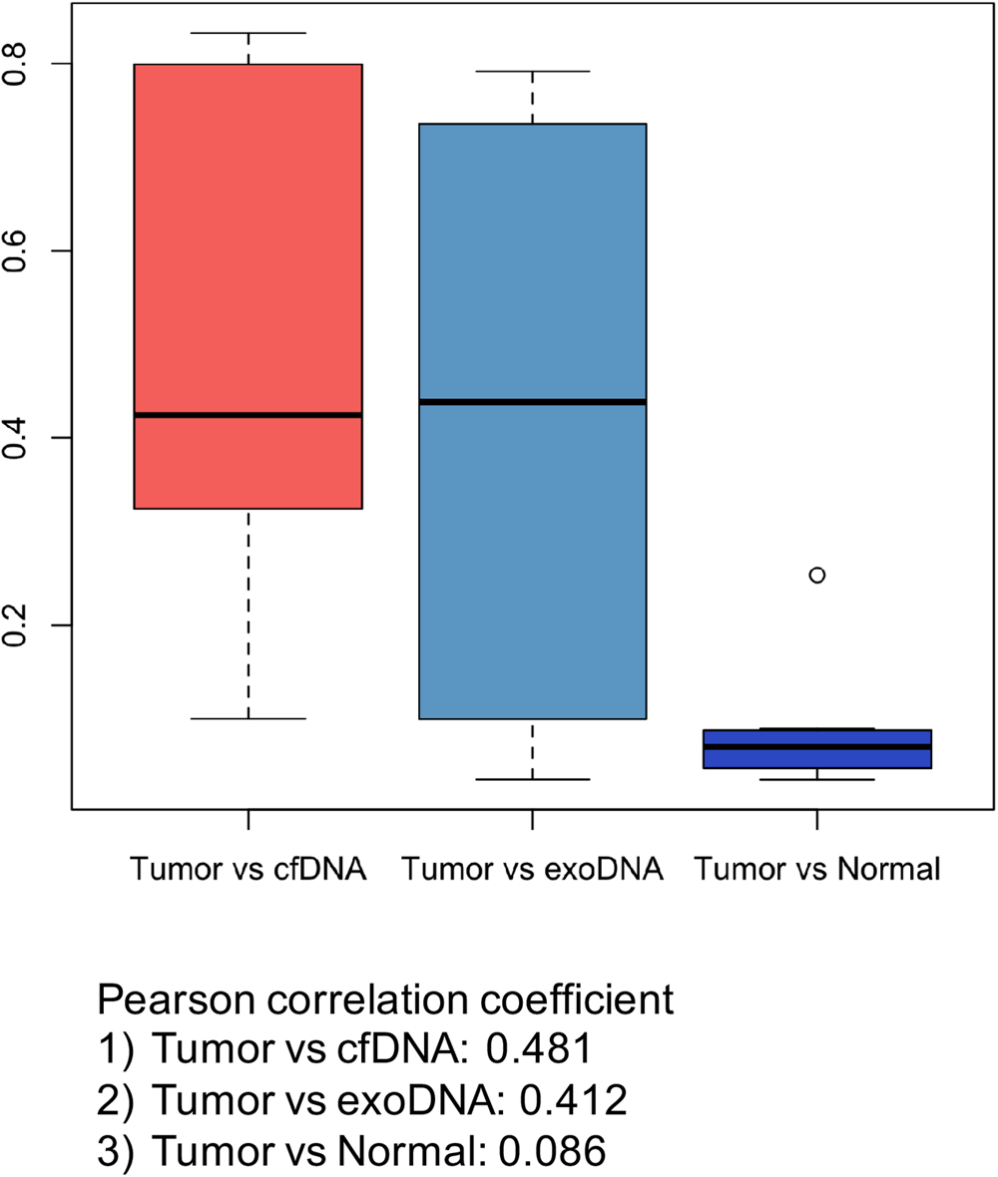
Similarities of copy number aberration between samples. While a mean Pearson correlation coefficient between tumor and normal samples was 0.086, those between tumor and cfDNA, and tumor and exoDNA, were 0.481 and 0.412, respectively.

For copy number analysis from sWGS data, we used QDNAseq as previously described^21^. This method provides copy number analysis using less than 1X genome coverage. In our experiments, the mean depth was 0.6X. Copy number aberrations in 9 tumor samples and matched urinary cfDNA and exoDNA are summarized in Supplement Table S6. As shown in TCGA bladder data, we found amplification of MDM2, ERBB2, CCND1 and CCNE1, and deletion of CDKN2A, PTEN and RB1 in our samples. While normal blood samples did not show any copy number aberration, cfDNA and exoDNA samples presented a similar pattern of copy number aberrations with tumor samples, except for the BC1 patient (Fig. 3). We also statistically examined the copy number aberration similarity between samples. While the mean Pearson’s correlation coefficient between tumor and normal samples was 0.086, the correlation coefficients between tumor and cfDNA, and tumor and exoDNA were 0.481 and 0.412, respectively (Fig. 4).

## Discussion

In this study, we found that the genetic alterations in UBC were well reflected in both urinary cfDNA and exoDNA. Somatic mutations were identified in urinary DNA by target deep sequencing. Although our study mainly included advanced stage UBC, the mean AF of cfDNA in the analysis of somatic mutation was 54.5%, indicating an enriched burden of tumor DNA in cfDNA. In addition, both exoDNA and cfDNA had a similar pattern of CNVs with those of UBC tumor tissue, representing whole genomic DNA. While two patients showed poor correlation between tumor and urinary DNA (patients 1 and 3), we found that tumor and urinary cfDNA or exoDNA had a moderate correlation on average, and a strong correlation (Pearson correlation coefficient >0.7) was noted in 3 patients (patients 2, 5 and 9)^22^. This differs from the poor correlation observed between tumor and normal blood (Pearson correlation coefficient <0.3). In addition, we produced copy number data using QDNAseq algorithms and data from whole-genome sequencing were segmented by a bin size of 100 k in our analysis; thus, the copy number data of each sample consisted of 24,579 values. The accuracy of the Pearson correlation coefficient increases with increasing data volume, suggesting that the relationship between tumor and urinary cfDNA and exoDNA is significant despite the moderate correlation coefficient.

Urinary exoDNA could be another source for liquid biopsy to identify genetic alteration of tumors in UBC as well as in other malignancies. There is controversy around whether DNA is present within exosomes or on the surface of exosomes^16,18,23–25^. Nevertheless, exosome-derived DNA provides information on genomic alterations in the tumor and can be a useful source for liquid biopsy. In our study, urinary exosomes were isolated using a commercial kit, although this method could have disadvantages with regard to the purity of the exosomes compared to ultracentrifugation, the gold standard method for isolation of exosome. However, we proved the presence of exosomes in our study. Also, detection of large fragments of DNA extracted from urinary exosomes is consistent with results from previous studies^16,18,26^. Our results also demonstrated that the urinary exoDNA contained the information on genomic alterations of the tumor and analysis of urinary exoDNA provided similar results to that of urinary cfDNA. In addition, approximately 90% of urinary exoDNA matched the human reference genome, suggesting that exoDNA originates from host cells.

The QDNAseq algorithm is based on the depth of coverage method and is characterized by removing blacklisted regions and combined correction for sequence mapability and GC contents. Previous studies have demonstrated that copy number profiling of cfDNA was successfully performed with sWGS^27,28^. Matched reference samples are not always necessary for CNV analysis using the QDNAseq algorithm, indicating that this method could be properly utilized in liquid biopsy. While targeted deep sequencing provides limited information for the targeted genomic lesions, sWGS provides a wide range of data based on whole genome profiling of the tumor at a low cost. Therefore, sWGS may offer more chance to detect genomic alterations and analyze genomic characteristics of tumors. In particular, UBC is characterized by a high mutation frequency^20^, thus the target gene panel, which includes large number of genomic regions, carries the burden of high cost. Also, a patient with somatic mutations that rarely occur in UBC might be missed by a gene panel comprising a limited number of target regions. CNV analysis could be an alternative strategy for liquid biopsy in patients with a malignancy that has diverse mutation frequencies for multiple genes.

In our results, some mutations were identified only in urinary cfDNA or exoDNA (Fig. 2). This might be attributable to false positivity due to sequencing errors. However, approximately 50% of these mutations were clinically significant mutations identified in both cfDNA and exoDNA. Genomic discordance between primary tumor and cfDNA was also found in another study using digital PCR, a highly sensitive method for detecting mutations with very low allele frequencies^2^. Multiple UBCs are often present and genetic heterogeneity has been noted within and between tumors in same patients^29^. We speculate that these results are largely due to the intratumoral heterogeneity reflected in urinary DNA. Liquid biopsy has potential advantages in overcoming the limitations of conventional single-site biopsy. It also has important clinical implications in the field of precision medicine, as it can reflect the overall genetic profiles of cancers with multiple sub-clones.

Our study is not devoid of limitations. We used a commercial kit for isolation of exosome and exosome purity is not as high as that obtained when ultracentrifuge is used for isolation. ExoDNA extracted by our protocol could originate from other materials in the exosome pellet. Various methods have been examined to identify the best protocol for isolation of urinary exosomes^30,31^. However, these studies have mainly focused on miRNA or mRNA. Further studies are needed to determine how we can improve the method to extract host or tumor DNA from urinary exosomes. While we performed a pilot study on advanced cases due to higher burden of tumor DNA, it is also important to demonstrate our results in early-stage cases to develop the liquid biopsy with diagnostic purpose. In early-stage cancer, a large sample volume, a large amount of sequence data, and an ultrasensitive method are required to detect the extremely low quantity of tumor DNA^32,33^. With the decreasing cost of sequencing and the development of computational analysis for low frequency mutations, these limitations can be overcome. At the same time, it is necessary to develop a method to increase the proportion of tumor DNA in liquid biopsies. Extracellular vesicles with specific proteins or sizes may provide more tumor genetic information^34,35^. In addition, we included patients with UBC and a large amount of urinary cfDNA or exosomes might come directly from bladder tumor cells in the genitourinary tract. Genomic profiling of urinary cfDNA or exoDNA in other solid tumors can be helpful in determining whether urinary cfDNA or exoDNA from circulation could be utilized in liquid biopsy. Considering the instability and low quantity of cfDNA, exoDNA might be a more ideal source for liquid biopsy with urine in other solid tumors than cfDNA.

In conclusion, our proof-of-concept study demonstrated that both urinary cfDNA and exoDNA were representative of the entire human genome and allowed genomic profiling of UBC. Using cfDNA and exoDNA, we successfully identified somatic mutations and CNVs of UBC and we demonstrated that urinary exoDNA could be another source of liquid biopsy. In addition, CNV analysis using sWGS could be another strategy for liquid biopsy, providing data for whole genome regions at a low cost.

## Materials and Methods

### Study population and sample collection

We prospectively collected clinical information, tissue, blood and urine samples from patients undergoing surgical treatment for UBC. This study was approved by the Institutional Review Board of Ewha Medical Center (IRB No. 2017-02-030-001) and all patients provided informed consent for tissue banking and genetic testing. All study protocol was carried out in accordance with the Declaration of Helsinki Guidelines. Blood and urine samples were collected prior to surgery and tumor tissue was stored as fresh frozen after surgery. Donated tumor tissues were reviewed by a genitourinary pathologist (S.H. Park) and analyzed for tumor characteristics and tumor contents. Tumor samples with a tumor content greater than 60% were considered suitable for genetic analysis. Samples consisted of 9 tumor tissues and 9 matched blood samples and 9 urine samples. Urine samples were divided into two aliquots for extraction of cfDNA and exoDNA in each patient.

### Urinary exosome isolation and exoDNA extraction

For the isolation of urinary exosomes, we used a commercial exosome precipitation reagent, ExoQuick-TC (System Biosciences, Mountain View, CA). Exosomes were isolated from 10–20 ml of urine according to the manufacturer’s instructions. Briefly, the urine samples were centrifuged at 2,000 × g at 4 °C for 15 minutes to remove cells and the corresponding amount of reagents were added. The mixtures were incubated at 4 °C overnight, then centrifuged at 1,500 × g for 30 minutes. After aspiration of supernatants, residuals were spin down at 1,500 × g for 5 minutes. Pellets were resuspended in 200 μl of phosphate-buffered saline.

To determine the size distribution of exosome pellets, a Nanosight LM10 system (Nanosight, Amesbury, UK) was used with Nanoparticle Tracking Analysis (NTA) software (ver 3.0 0064). Microscopic imaging was also performed to characterize the pellets with electron microscopy. For electron microscopy, samples were placed on 200 mesh Formvar-coated copper grids treated with poly-L-lysine for 1 hour. Excess samples were blotted with filter paper and then negatively stained with Millipore paper-filtered aqueous 1% uranyl acetate for 2 minutes. The stain was blotted dry from the grids with filter paper, and samples were allowed to dry. Samples were then examined with a H-7650 transmission electron microscope (HITACHI, Ibaraki-ken, Japan) at an accelerating voltage of 80 kV. Digital images were obtained using the soft imaging system GmbH (Munster, Germany). To validate the presence of exosomes, Western blot analysis was performed using Alix and TSG101, common exosomal markers. Prepared exosomes were lysed with RIPA buffer [50 mM Tris-HCl (pH 8.0), 150 mM NaCl, 0.1% SDS, 0.5% deoxycholate, 1% NP-40, and protease/phosphatase inhibitors (Sigma-Aldrich)]. Lysates were separated by SDS-PAGE, transferred onto a PVDF membrane, and then incubated with anti-Alix (Millipore #ABC40) and anti-TSG101 (Abcam #ab83) antibodies and horseradish peroxidase (HRP)-conjugated secondary antibodies (Cell Signaling Technology). Protein bands were visualized with PicoEPD (Enhanced Peroxidase Detection) Western Reagent Kit (Elpis-Biotech, Daejeon, Korea). The size distribution graph showed the presence of exosome-sized vesicles and electron microscopy confirmed the presence of exosomes. Alix and TSG101 were also confirmed in the Western blot analysis (Supplementary Fig. S1).

### Extraction and characterization of DNA

DNA from tissue and blood samples was extracted using QIAamp DNA Minikit (Qiagen, Valencia, CA). ExoDNA was extracted from urinary exosome using the same DNA extraction kit. The extraction of cfDNA from urine was performed using a MagMax Cell-Free DNA Isolation Kit (Thermo Fisher Scientific), which uses magnetic bead technology. CfDNA was extracted from 2–4 ml of urine according to manufacturer’s instruction. The concentration and integrity of the extracted cfDNA and exoDNA from urine were determined using the Agilent 2200 TapeStation (Agilent Technologies, Santa Clara, CA) and the associated High Sensitivity D1K ScreenTape (Agilent Technologies, Santa Clara, CA).

### Library preparation and sequencing

DNA from tissue and blood was sheared into 300-bp fragments on average with a Bioruptor® Pico (Diagenode diagnostics, Belgium). ExoDNA was sheared into 180-bp fragments, which was similar to the size of cfDNA. The fragmentation step was not performed in cfDNA samples. A sequencing library was constructed with Celemics library preparation kit and the prepped libraries were enriched with Illumina index primers and KAPA HiFi HotStart PCR kit. Target gene capture was performed with customized capture probes and a Celemics target capture kit. For the target capture kit, we selected 9 genes, which were frequently mutated in UBC, based on previous studies^36–39^. These genes included ARID1A, PIK3CA, FGFR3, HRAS, KMT2D, RB1, TP53, KDM6A and STAG2. The captured libraries were enriched with KAPA HiFi HotStart PCR kit (Kapa Biosystems, Boston, USA). Next-generation sequencing was performed using the Illumina NextSeq. 500 platform (Illumina, San Diego, CA) with 2 × 150 bp paired-end run. For sWGS, the libraries were directly sequenced by Illumina HiSeq. 2500 platform (Illumina, San Diego, CA). For target capture sequencing, we performed deep sequencing on urinary cfDNA and exoDNA. However, sWGS was performed with a similar sequencing depth through all samples. Sequencing depth and coverage are described in Supplement Table S1. Sequencing data are accessible in the Sequence Read Archive (accession number [SRR139143]).

### Alignment and somatic variant calling

Burrows-Wheeler aligner (BWA; version 0.7.10) was used to align reads to the UCSC human genome (GRCh37/hg19). The sequence alignment map (SAM) file was converted to BAM format using SAMtools (version 1.1). The Picard tool (version 1.115) was used to sort and remove duplications. GATK-Lite (version 2.3.9) was used to perform indel realigning and base quality score re-calibration. For targeted sequencing data, SAMtools mpileup was used to create an mpileup file with a minimum base-quality of 17 and Varscan (version 2.4.0) was used for mutation and indel call. The threshold of allele frequency (AF) less than a 5% was used to filter out false-positive mutations. Mutation or indel calls in tumor samples without a call in the matched normal blood sample was defined as a somatic mutation. For somatic mutation calls of germline heterozygosity, AF of 20% greater than those of normal blood sample was defined as a mutation. False-positive indels were removed by manually reviewing each indel with the Integrated Genome Viewer (IGV version 2.3.0.). Nonsynonymous, stop-gain SNV and frameshift indel were considered clinically actionable mutations.

### Detection of copy number variation

CNV was analyzed by QDNAseq (version 1.12.0) after alignment of sWGS data^21^. Briefly, we used 100 kb bins for allocation of sequence reads. Correction for GC-mapability and exclusion of problematic regions was subsequently performed to obtain high-quality copy number information from the sWGS data. The median-normalized log_2_-transformed read count was calculated and CNV calls with >0.3 or <-0.3 log_2_ values were considered to indicate significant amplification or deletion, respectively.

### Data analysis and statistics

We analyzed whether genetic alterations in tumor samples were detected in urinary cfDNA or exoDNA. For somatic mutation analysis, we also investigated the false positivity, which indicates somatic mutation that is only identified in cfDNA or exoDNA. To measure similarity between copy number profiles of tumor tissue and urinary cfDNA or exoDNA, the Pearson’s correlation efficient was calculated. Statistical analysis was performed using R, version 3.2.5 (http://www.r-project.org).

## Electronic supplementary material

Supplementary Information

Supplementary Table S4-6
