# Supplementary material for: Urinary Exosomal and cell-free DNA Detects Somatic Mutation and Copy Number Alteration in Urothelial Carcinoma of Bladder
Source: Sci Rep. 2018 Oct 2;8:14707. doi: 10.1038/s41598-018-32900-6 (PMC6168539; doi:10.1038/s41598-018-32900-6)
Supplement: Supplementary file 1 — Supplementary Information [file 41598_2018_32900_MOESM1_ESM.pdf]

Supplementary Information

Urinary Exosomal and cell-free DNA Detects Somatic Mutation and Copy Number Alteration in Urothelial Carcinoma of Bladder

Dong Hyeon Lee[1], Hana Yoon[1], Sanghui Park[2], Jeong Seon Kim[3], Young-Ho Ahn[3]

Kihwan Kwon[4], Donghwan Lee[5], Kwang Hyun Kim[1]

[1]Department of Urology, Ewha Womans University College of Medicine, Seoul, Korea

[2] Department of Pathology, Ewha Womans University College of Medicine, Seoul, Korea

[3] Department of Molecular Medicine, Ewha Womans University College of Medicine, Seoul, Korea

[4]Department of Cardiology, Ewha Womans University College of Medicine, Seoul, Korea

[5]Department of Statistics, Ewha Womans University, Seoul, Korea

CORRESPONDENCE:

Kwang Hyun Kim

Department of Urology, Ewha Womans University College of Medicine

1071, Anyangcheon-ro, Yangcheon-gu, Seoul 158-710, Korea

Tel: 82-2-2228-5873

Fax: 82-2654-3682

E-mail: khkim.uro@gmail.com

Supplementary Figure 1. Characteristics of exosomes isolated from urine. (A) Electron microscopic image identifies vesicles with a size in the range of 100-150 nm. (B) The size distribution graph measured by nanoparticle tracking system shows the presence of exosome sized vesicles. (C) Western blot of exosomal marker (Alix and TSG101).

(A)

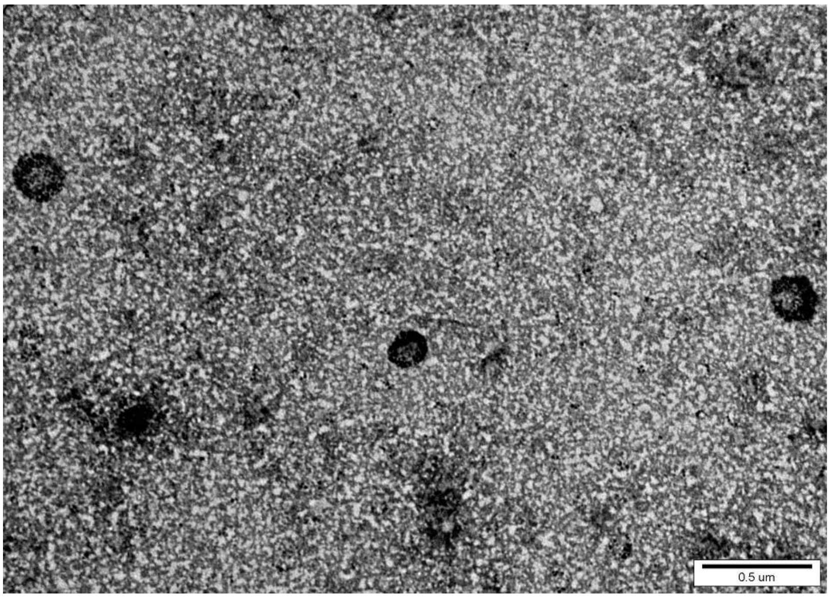

(B)

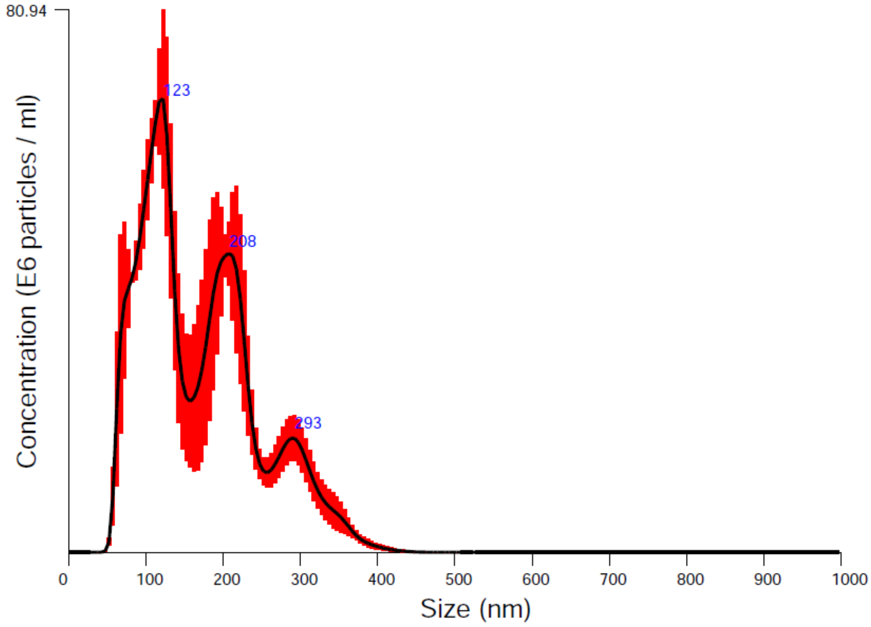

(C)

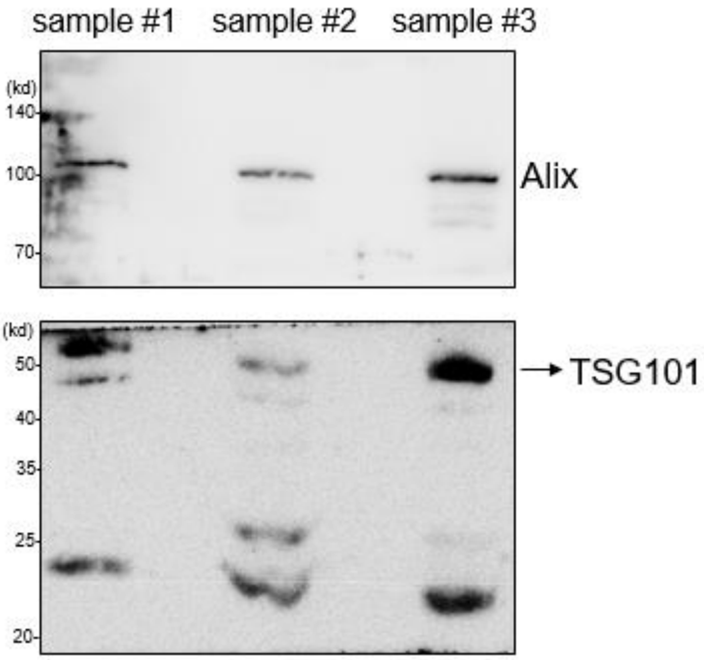

sample #1
sample #2
sample #3
(kd)
140
100
70
Alix
(kd)
50
40
35
25
20
TSG101

Supplementary Figure 2. Integrity of urinary cell free DNA (cfDNA) and exosomal DNA (exoDNA) was analyzed using Agilent 2200 TapeStation. While cfDNA was highly fragmented with mostly size of 150-180 bp (A), exoDNA contains a large fragmented DNA compared cfDNA (B).

(A)

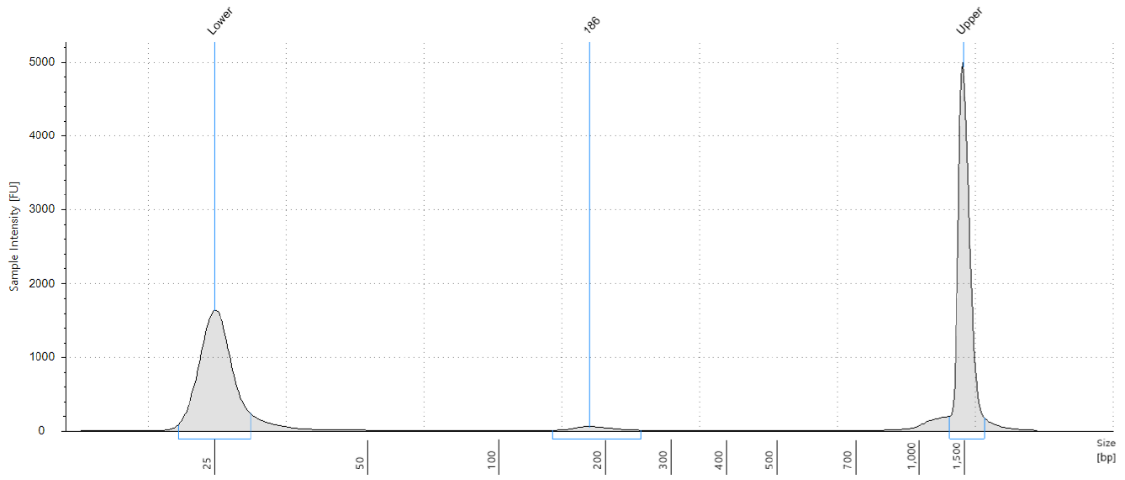

(B)

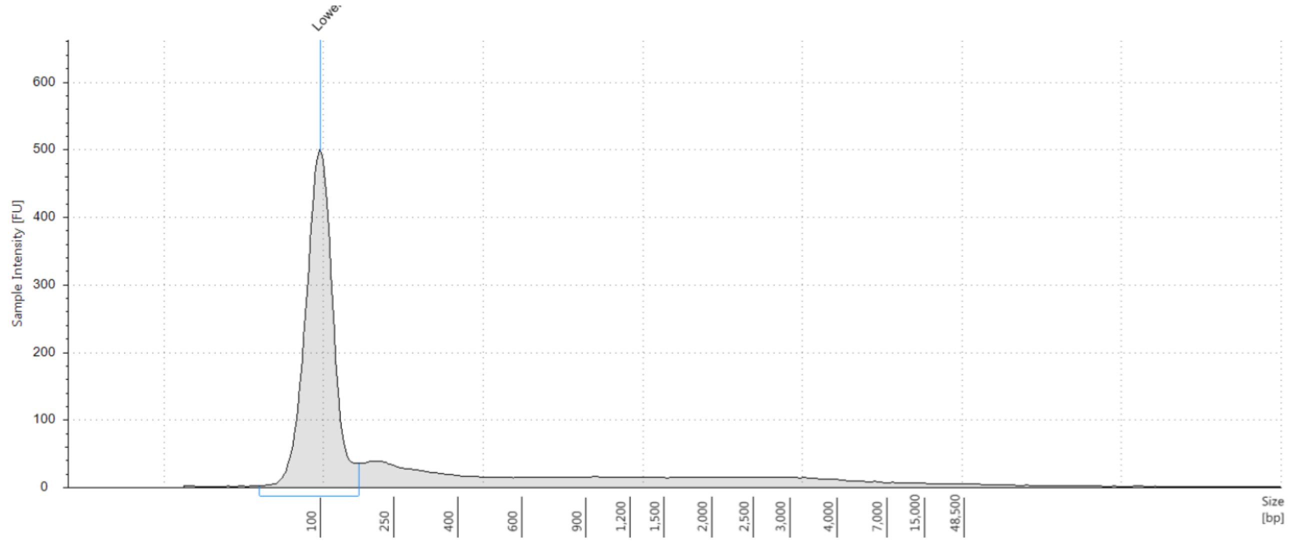

Supplementary Figure 3. Copy number variation profiles analyzed by shallow whole genome sequencing in 9 patients with urinary bladder cancer.

Patient #1

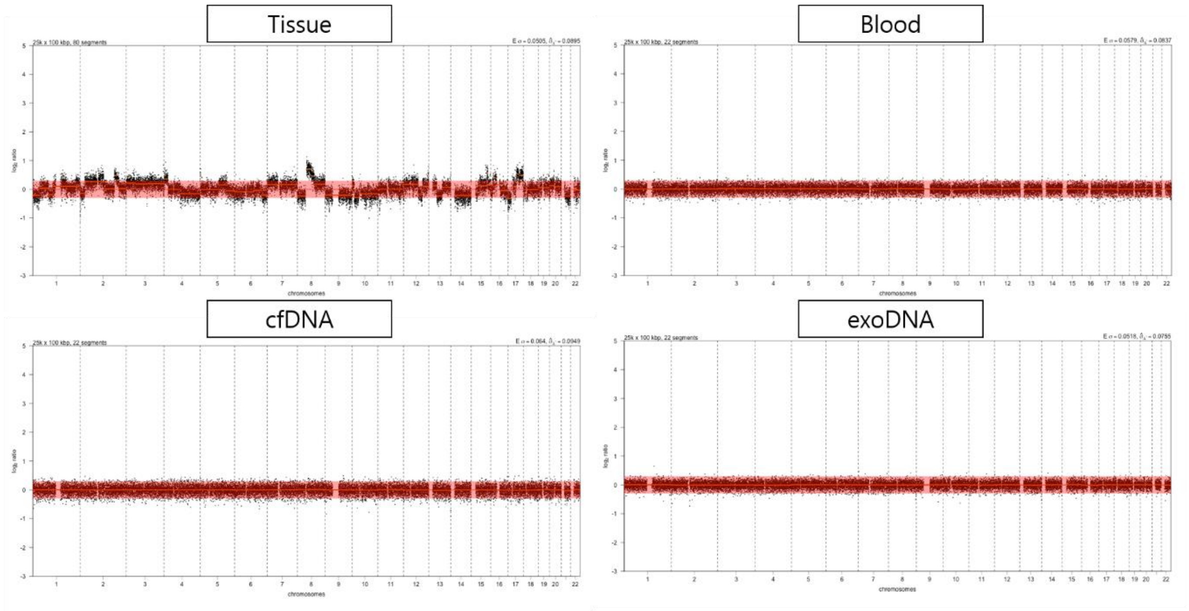

Patient #2

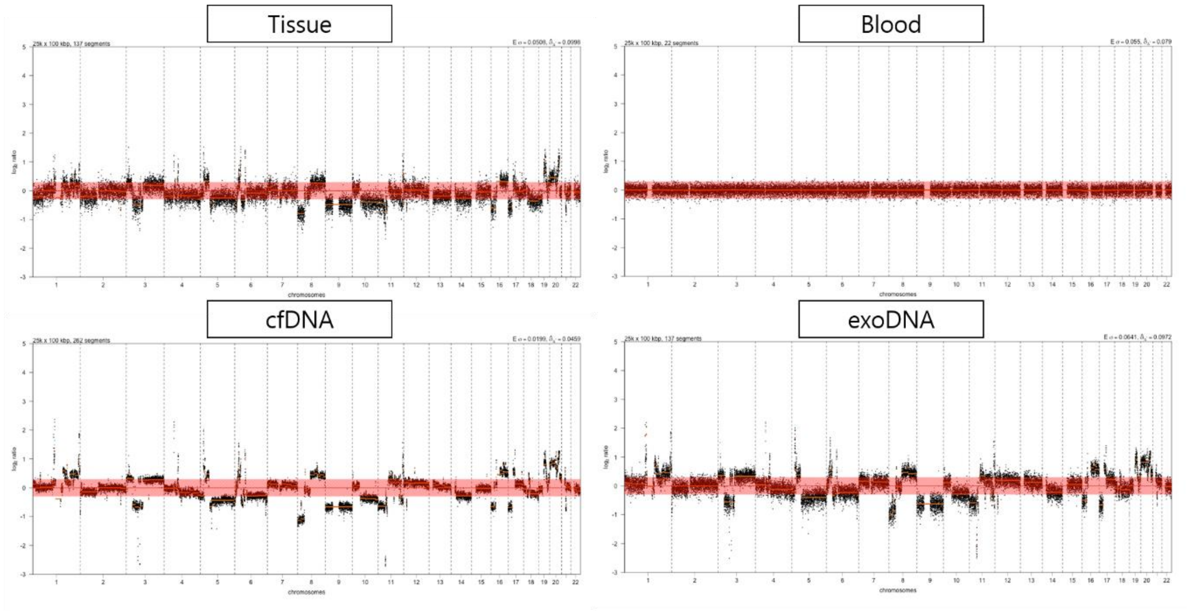

Patient #3

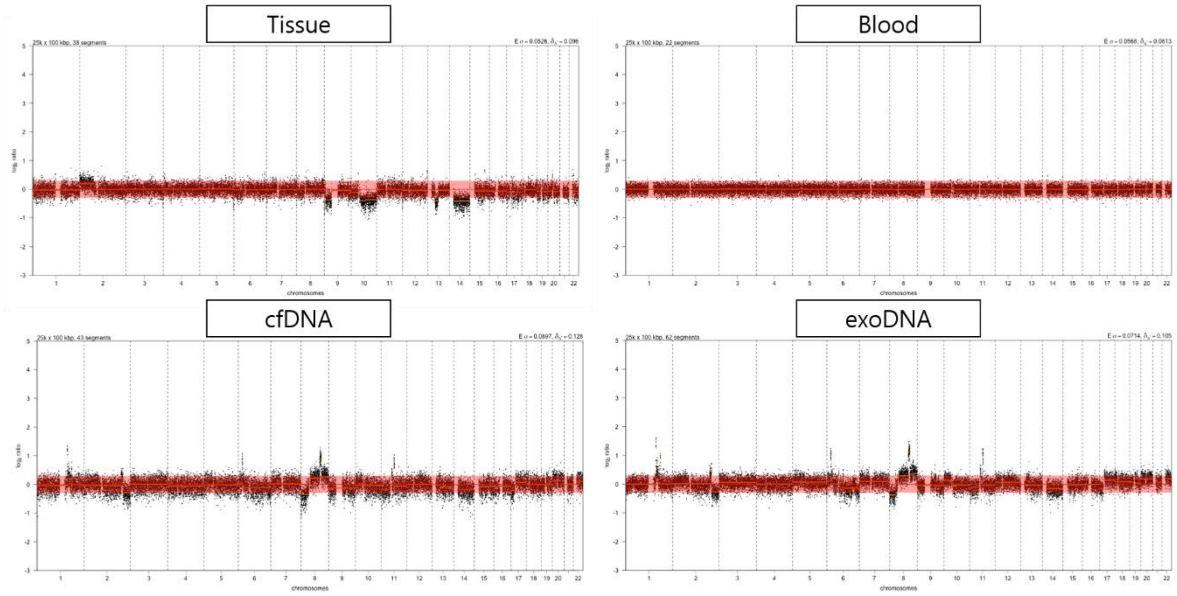

Patient #4

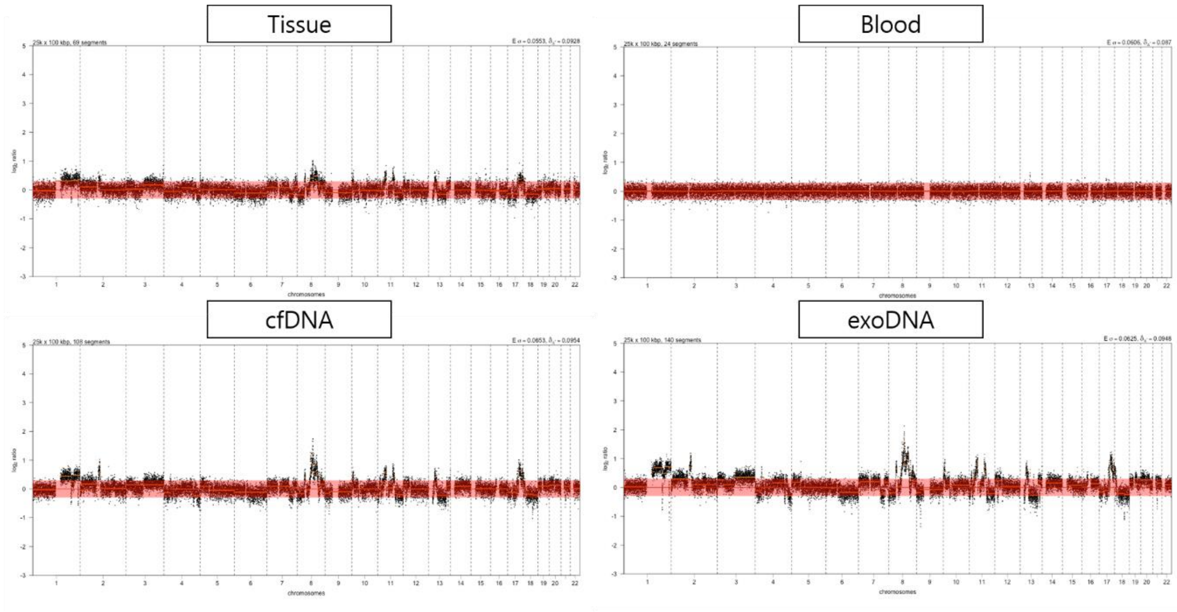

Patient #5

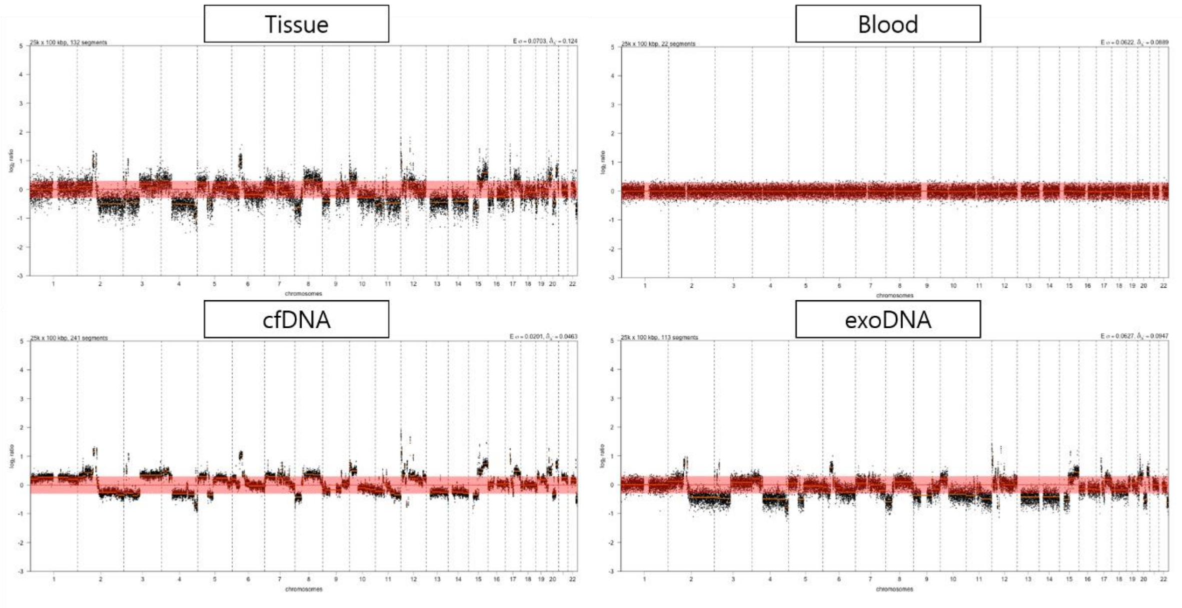

Patient #6

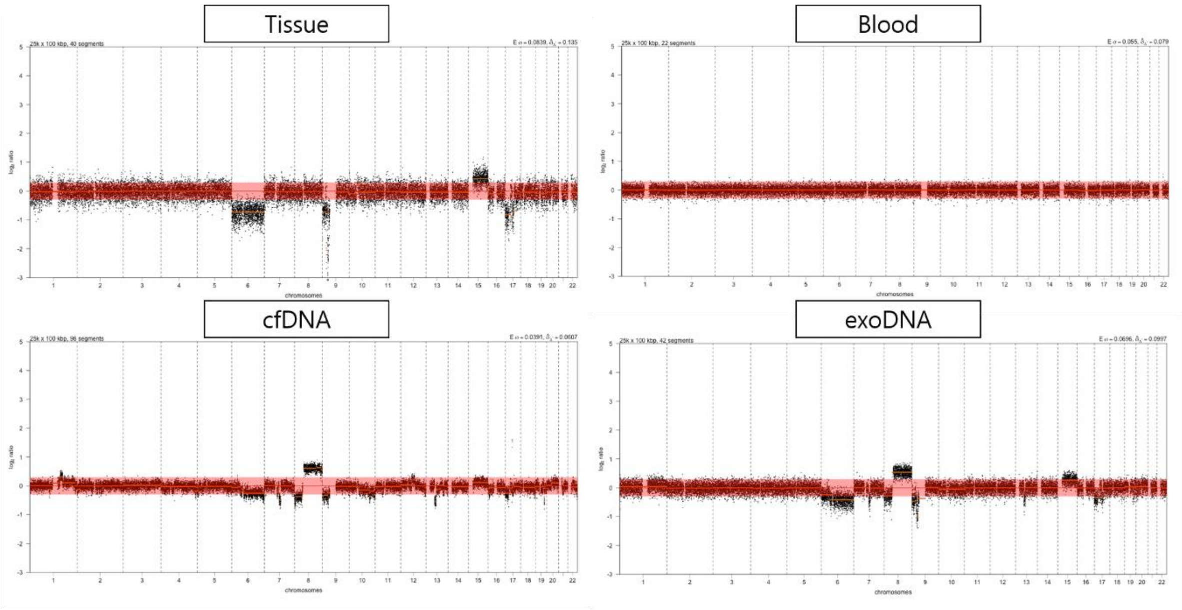

Patient #7

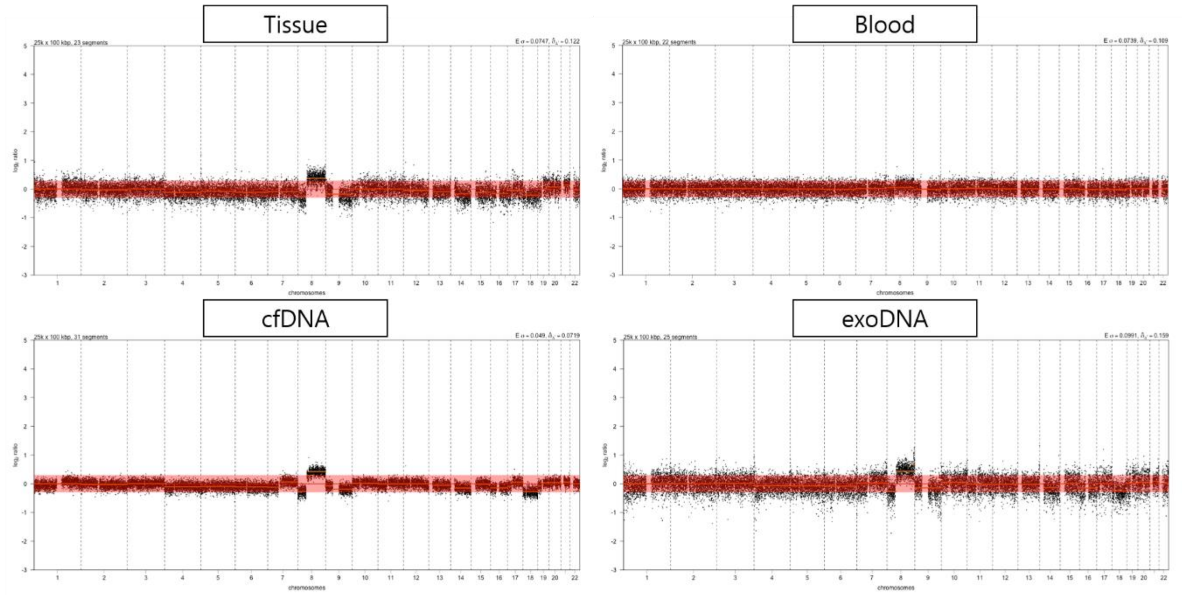

Patient #8

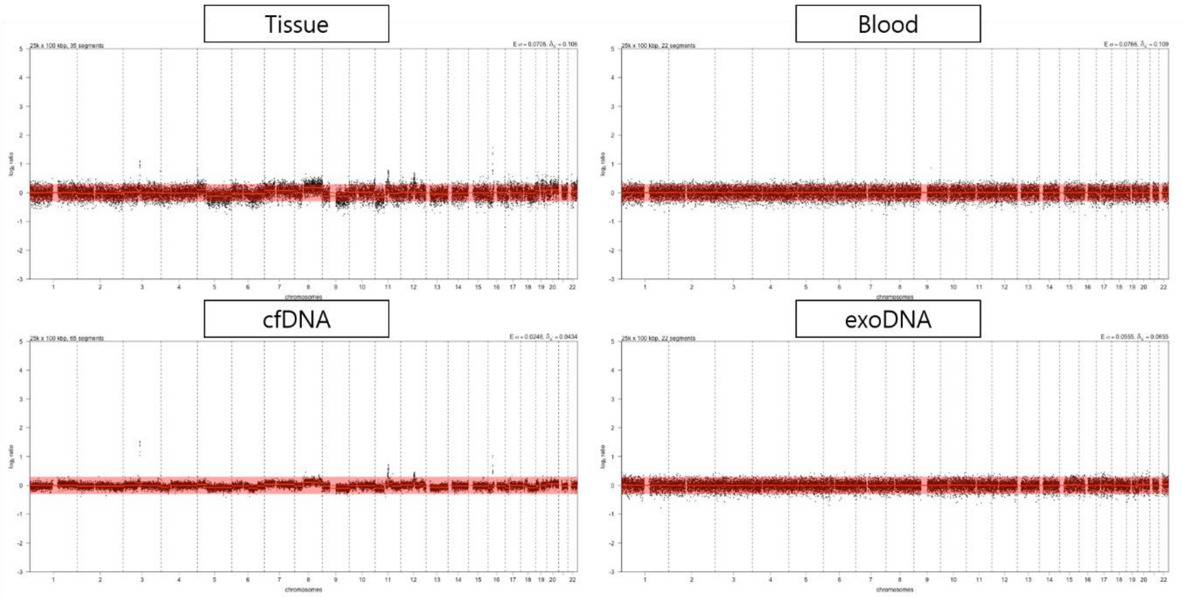

Patient #9

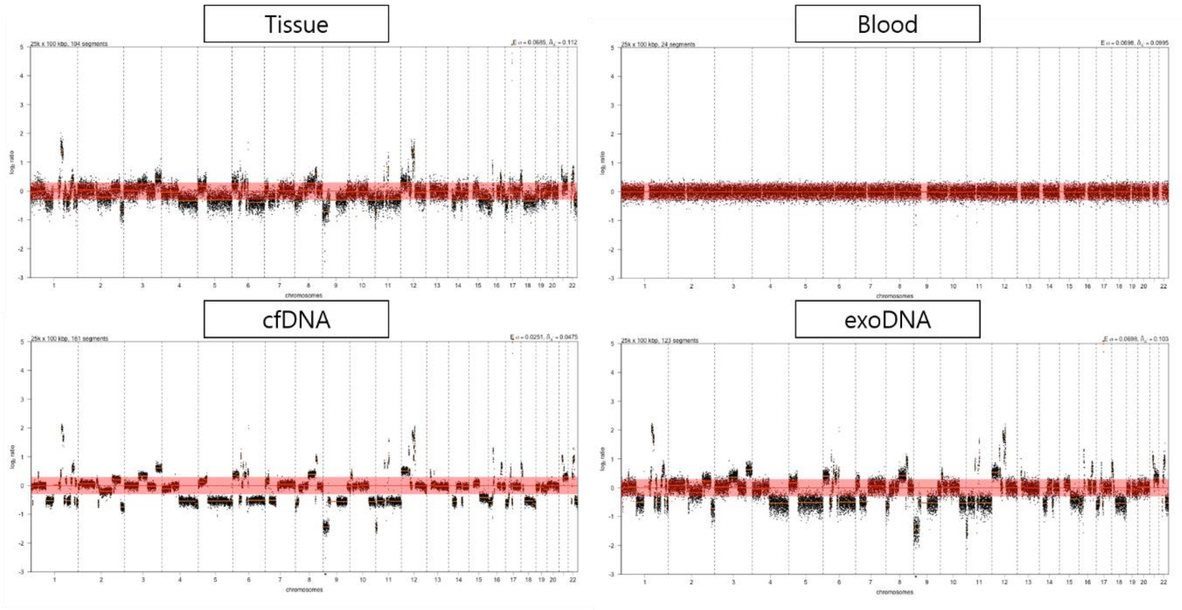

Supplemantary Table S1. Coverage and sequencing depth of target capture sequencing and ldWGS

Target capture sequencing							
Sample		Depth	1X (%)	10X (%)	20X (%)	50X (%)	100X (%)
BC1	Tumor	642.4194	99.71%	99.47%	99.12%	98.29%	96.25%
	Normal	524.7613	99.55%	99.28%	98.95%	98.02%	96.02%
	cfDNA	1902.773	99.71%	99.62%	99.55%	99.24%	98.94%
	exoDNA	1126.985	99.76%	99.67%	99.58%	99.45%	99.15%
BC2	Tumor	623.6012	99.84%	99.61%	99.47%	98.89%	96.47%
	Normal	441.468	99.66%	99.24%	98.87%	97.70%	95.44%
	cfDNA	1708.467	99.69%	99.49%	99.28%	99.05%	98.43%
	exoDNA	555.1018	99.96%	99.71%	99.64%	99.50%	99.08%
BC3	Tumor	688.4147	99.74%	99.68%	99.53%	98.90%	97.03%
	Normal	473.4652	99.72%	99.34%	99.21%	97.91%	95.71%
	cfDNA	1109.807	99.62%	99.49%	99.28%	99.02%	98.56%
	exoDNA	636.1312	99.74%	99.65%	99.62%	99.42%	98.98%
BC4	Tumor	558.1419	99.64%	99.27%	98.71%	97.81%	95.64%
	Normal	220.4392	99.69%	98.89%	98.34%	95.42%	87.03%
	cfDNA	1583.164	99.64%	99.43%	99.28%	99.00%	98.18%
	exoDNA	1716.73	99.97%	99.74%	99.72%	99.63%	99.49%
BC5	Tumor	571.6812	99.74%	99.33%	99.04%	98.06%	94.92%
	Normal	131.1689	99.53%	98.09%	96.77%	89.60%	67.92%
	cfDNA	1921.558	99.71%	99.65%	99.59%	99.33%	99.07%
	exoDNA	1150.288	99.94%	99.73%	99.70%	99.57%	99.51%
BC6	Tumor	841.9534	99.74%	99.70%	99.53%	99.15%	98.01%
	Normal	804.756	99.76%	99.70%	99.61%	99.26%	98.16%
	cfDNA	1992.206	99.76%	99.72%	99.70%	99.49%	99.25%
	exoDNA	4909.805	99.99%	99.75%	99.74%	99.72%	99.62%
BC7	Tumor	834.4549	99.75%	99.68%	99.54%	99.27%	98.19%
	Normal	739.265	99.76%	99.71%	99.61%	99.32%	98.16%
	cfDNA	1399.131	99.77%	99.67%	99.59%	99.52%	99.06%
	exoDNA	102.9424	99.72%	98.98%	96.15%	55.78%	31.26%
BC8	Tumor	612.1861	99.67%	99.58%	99.37%	98.97%	97.56%
	Normal	688.5387	99.97%	99.65%	99.50%	99.06%	97.62%
	cfDNA	2483.192	99.76%	99.72%	99.61%	99.51%	99.37%
	exoDNA	321.41	99.73%	99.64%	99.55%	99.02%	96.87%
BC9	Tumor	45.09224	99.39%	93.99%	84.14%	37.74%	3.49%
	Normal	743.8648	99.76%	99.69%	99.60%	99.16%	98.13%
	cfDNA	3250.546	99.77%	99.75%	99.72%	99.64%	99.53%
	exoDNA	4313.715	99.77%	99.74%	99.73%	99.69%	99.61%

low depth whole genome sequencing					
Sample		Mapped read ratio	Depth	1X (%)	10X (%)
BC1	Tumor	97.30%	0.50	30.8150%	0.0040%
	Normal	95.41%	0.39	25.2270%	0.0040%
	cfDNA	88.39%	0.29	17.5810%	0.0030%
	exoDNA	85.82%	0.44	24.6750%	0.0050%
BC2	Tumor	97.48%	0.50	30.6320%	0.0030%
	Normal	95.34%	0.43	26.6720%	0.0040%
	cfDNA	93.09%	3.17	79.7670%	1.8200%
	exoDNA	92.43%	0.29	16.8620%	0.0030%
BC3	Tumor	96.84%	0.46	28.5740%	0.0040%
	Normal	94.98%	0.40	26.0110%	0.0040%
	cfDNA	90.34%	0.13	7.9690%	0.0020%
	exoDNA	91.76%	0.24	14.3770%	0.0030%
BC4	Tumor	96.74%	0.42	26.7600%	0.0030%
	Normal	94.64%	0.35	22.4330%	0.0040%
	cfDNA	93.73%	0.30	19.2130%	0.0030%
	exoDNA	94.35%	0.32	19.3830%	0.0030%
BC5	Tumor	97.87%	0.26	17.0730%	0.0010%
	Normal	95.19%	0.33	22.0300%	0.0030%
	cfDNA	92.71%	3.01	78.9470%	1.3100%
	exoDNA	94.56%	0.32	20.1010%	0.0040%
BC6	Tumor	97.39%	0.18	13.8697%	0.0022%
	Normal	94.98%	0.27	19.5561%	0.0040%
	cfDNA	92.81%	0.78	44.7637%	0.0134%
	exoDNA	94.29%	0.26	18.5949%	0.0039%
BC7	Tumor	96.55%	0.23	17.1512%	0.0027%
	Normal	95.43%	0.23	17.5359%	0.0032%
	cfDNA	90.92%	0.51	32.8704%	0.0073%
	exoDNA	93.62%	0.11	6.8556%	0.0028%
BC8	Tumor	94.47%	0.26	18.6553%	0.0035%
	Normal	95.18%	0.22	16.3368%	0.0028%
	cfDNA	93.53%	2.01	77.0901%	0.2952%
	exoDNA	95.28%	0.35	20.1124%	0.0035%
BC9	Tumor	97.02%	0.27	19.9945%	0.0056%
	Normal	94.86%	0.26	19.1884%	0.0038%
	cfDNA	91.10%	1.97	74.0020%	0.7459%
	exoDNA	91.84%	0.25	16.7971%	0.0138%

Supplementary Table S2. Clinicopathological characteristics of patients included in this study

	Gender	Age (year)	Surgery	Stage	Grade
BC1	male	48	Radical cystectomy	T4N2	high
BC2	male	66	Radical cystectomy	T2N0	high
BC3	male	60	Radical cystectomy	T3N2	high
BC4	male	71	Radical cystectomy	T3N2	high
BC5	male	66	Radical cystectomy	T2N2	high
BC6	male	46	Radical cystectomy	T2N0	high
BC7	male	58	Radical cystectomy	T3N2	high
BC8	male	41	Radical cystectomy	T3N2	high
BC9	male	68	Radical cystectomy	T2N0	high

Supplementary Table S3. The concentration and yeild of urinary DNA

	cfDNA		
	urine volume (ml)	DNA amount (ng)	concentration (ng/ml)
BC1	2	73.2	36.6
BC2	4	38.1	9.525
BC3	4	23.1	5.775
BC4	2	50.1	25.05
BC5	4	24.5	6.125
BC6	4	83.91	20.9775
BC7	4	20.4	5.1
BC8	4	111.2	27.8
BC9	4	87.9	21.975

	exoDNA		
	urine volume (ml)	DNA amount (ng)	concentration (ng/ml)
BC1	10	31.5	3.15
BC2	20	141.3	7.065
BC3	10	47.5	4.75
BC4	10	84.4	8.44
BC5	10	77.3	7.73
BC6	10	48.6	4.86
BC7	10	25	2.5
BC8	10	5.58	0.55
BC9	10	320	32

Supplementary Table S4-S6

: Due to size and amount of data presented in the supplementary tables, we included Tables S4-S6 in another file as the Supplementary Dataset.

Supplementary Table S7. Pearson correlation coefficient between tumor and normal blood, cfDNA and exoDNA.

	Tumor vs. Normal	Tumor vs. cfDNA	Tumor vs. exoDNA
BC1	0.09	0.12	0.04
BC2	0.07	0.80	0.74
BC3	0.09	0.10	0.10
BC4	0.09	0.53	0.57
BC5	0.06	0.83	0.75
BC6	0.04	0.32	0.44
BC7	0.25	0.38	0.22
BC8	0.04	0.42	0.07
BC9	0.05	0.83	0.79
